# Impact of Chronic Inflammation, Assessed by hs-CRP, on the Association between Red Cell Distribution Width and Arterial Cardiovascular Disease: The Tromsø Study

**DOI:** 10.1055/s-0038-1651523

**Published:** 2018-05-16

**Authors:** Jostein Lappegård, Trygve S. Ellingsen, Kristian Hindberg, Ellisiv B. Mathiesen, Inger Njølstad, Tom Wilsgaard, Maja-Lisa Løchen, Sigrid K. Brækkan, John-Bjarne Hansen

**Affiliations:** 1 K.G. Jebsen Thrombosis Research and Expertise Center (TREC), Department of Clinical Medicine, UiT The Arctic University of Norway, Tromsø, Norway; 2 Brain and Circulation Research Group, Department of Clinical Medicine, UiT The Arctic University of Norway, Tromsø, Norway; 3 Department of Neurology and Neurophysiology, University Hospital of North Norway, Tromsø, Norway; 4 Department of Community Medicine, UiT The Arctic University of Norway, Tromsø, Norway; 5 Division of Internal Medicine, University Hospital of North Norway, Tromsø, Norway

**Keywords:** epidemiology, myocardial infarction, stroke, blood cells, risk factors

## Abstract

Red cell distribution width (RDW), a measure of variability in size of circulating erythrocytes, is associated with arterial cardiovascular disease (CVD), but the underlying mechanism remains unclear. We aimed to investigate the impact of chronic inflammation as measured by high-sensitivity C-reactive protein (hs-CRP) on this relationship, and explore whether RDW could be a mediator in the causal pathway between inflammation and arterial CVD. Baseline characteristics, including RDW and hs-CRP, were obtained from 5,765 individuals attending a population-based cohort study. We followed up participants from inclusion in the fourth survey of the Tromsø Study (1994/1995) until December 31, 2012. Multivariable Cox-regression models were used to calculate hazard ratios (HR) with 95% confidence intervals (CI) for incident myocardial infarction (MI) and ischemic stroke across quintiles of hs-CRP and RDW. Subjects with hs-CRP in the highest quintile had 44% higher risk of MI (HR: 1.44, 95% CI: 1.14–1.80), and 64% higher risk of ischemic stroke (HR: 1.64, 95% CI: 1.20–2.24) compared with subjects in the lowest quintile. RDW mediated 7.2% (95% CI: 4.0–30.8%) of the association between hs-CRP and ischemic stroke. Subjects with RDW in the highest quintile had 22% higher risk of MI (HR: 1.22, 95% CI: 0.98–1.54) and 44% higher risk of ischemic stroke (HR: 1.44, 95% CI: 1.06–1.97) compared with subjects in the lowest quintile. These risk estimates were slightly attenuated after adjustments for hs-CRP. Our findings suggest that chronic inflammation is not a primary mechanism underlying the relationship between RDW and arterial CVD.

## Introduction

Red blood cell distribution width (RDW) is a measure of the variability in size of circulating erythrocytes, and is reported by most automated blood cell counters. 1 In combination with mean corpuscular volume (MCV), RDW has traditionally been used in the differential diagnosis of anemia. 2 Various causes of premature release of red blood cells may lead to alterations in the RDW. 3 4 5

Growing evidence supports a relationship between RDW and arterial cardiovascular disease (CVD). In two large population-based cohorts, RDW was associated with future risk of incident MI 6 7 and ischemic stroke. 8 9 However, the mechanisms behind these relationships are not clear.

Atherosclerosis, the principle cause of myocardial infarction (MI) and an important cause of ischemic stroke, 10 is widely recognized as an inflammatory disease. 11 12 C-reactive protein (CRP), an acute phase reactant synthesized in response to signals from macrophages and T cells during inflammation (e.g., interleukin-6), 13 is a predictor of MI and stroke. 14 15 16 17 Inflammation affects red blood cell life span and modulates the effect of erythropoietin on the erythropoiesis, and can thereby affect RDW. 18 Studies have reported an association between RDW and CRP, erythrocyte sedimentation rate (ESR), and interleukin-6. 19 20 21 22

**Fig. 1:**
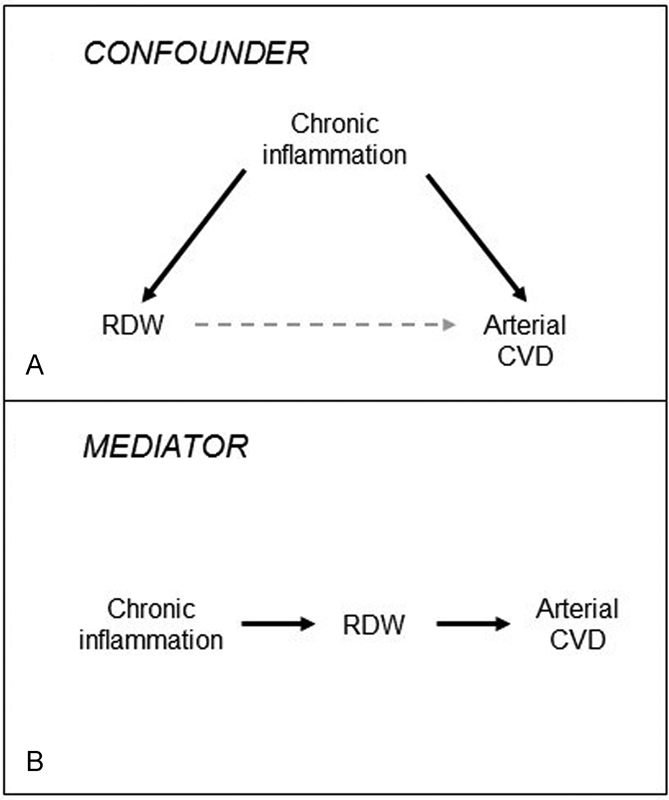
Potential associations between chronic inflammation, red cell distribution width (RDW), and risk of cardiovascular disease (CVD). ( **A** ) Illustration of how chronic inflammation could act as a confounder of the association, affecting both RDW and risk of CVD. ( **B** ) Illustration of how RDW could act as a mediator in the causal pathway between chronic inflammation and arterial CVD.

Since chronic inflammation is associated with increased risk of arterial CVD, and RDW is associated with several markers of chronic inflammation, it has been hypothesized that the apparent association between RDW and risk of arterial CVD could be explained by chronic inflammation. On one hand, chronic inflammation could merely serve as a confounder for the relationship between RDW and arterial CVD ( Fig. 1A ). Alternatively, if RDW is causally associated with arterial CVD (e.g., through mechanisms of augmented erythrocyte aggregability or altered blood viscosity), it could act as mediator in the pathway between chronic inflammation and CVD ( Fig. 1B ).

Despite the indications that chronic inflammation may influence the association between RDW and arterial CVD, this hypothesis has not been thoroughly investigated. Therefore, we set out to study whether the association between RDW and arterial CVD was confounded by chronic inflammation (assessed by hs-CRP), or whether chronic inflammation was associated with changes in RDW (mediator) that directly could lead to increased risk of arterial CVD.

## Materials and Methods

### Study Population

The fourth survey of the Tromsø study (Tromsø 4) was conducted in 1994–1995, and all inhabitants in the municipality of Tromsø, Norway, aged 25 or older, were invited to participate. A total of 27,158 individuals attended the first visit, yielding a participation rate of 77%. Following this, all participants aged 55 to 74 years, as well as 5 to 8% randomly selected samples from each of the other 5-year age intervals (between 25–54 years and 75–85 years), were offered a follow-up visit (1994–1995). At this second visit, the participants underwent a more thorough examination, and additional measurements including high-sensitivity CRP (hs-CRP) were performed. All subjects attending the second visit of Tromsø 4 were enrolled in the present study ( *n* = 6,834; 76% of those eligible). The study population has been described in detail elsewhere. 23 We excluded subjects lacking measurements of RDW ( *n* = 169) and hs-CRP ( *n* = 118), subjects with a diagnosis of MI ( *n* = 354) or stroke ( *n* = 166) prior to enrolment, as well as subjects migrating from Tromsø before the second visit ( *n* = 7). In addition, we excluded participants with hs-CRP levels >10 mg/L ( *n* = 255) at baseline to avoid influence of possible acute phase reactions. This left us with 5,765 participants in the present study. The Regional Committee for Medical and Health Research Ethics approved the study, and informed written consent was acquired from all participants.

### Baseline Measurements

Baseline information on each participant was obtained through self-administered questionnaires, blood samples, and physical examinations. Nonfasting blood samples were drawn from an antecubital vein into vacutainer tubes containing EDTA for anticoagulation (K3-EDTA 40 µL, 0.37 mol/L per tube). For the blood cell counts, including RDW, 5 mL of blood was analyzed within 12 hours in an automated blood cell counter (Coulter Counter; Coulter Electronics, Luton, UK). RDW was calculated by dividing the standard deviation (SD) of MCV with the MCV, then multiplying it by 100. 24 For preparation of serum, the blood sample was given a 1-hour respite at room temperature before centrifugation. Hs-CRP was measured by a particle-enhanced immunoturbidimetric assay on a Modular P auto-analyzer (Roche/Hitachi) using reagents from Roche Diagnostics GmbH, Mannheim, Germany. The lower detection limit for the assay was 0.03 mg/L, and all measurements below this threshold were set to 0.03 mg/L. Daily changes in the assay precision for values between 0.1 and 20 mg/L were less than 4%.

Trained personnel recorded blood pressure on the right upper arm with an automatic device (Dinamap Vital Signs Monitor; 1846, Critikon Inc., Tampa, Florida, United States). Starting with 2 minutes of rest in a seated position, three recordings with 2-minute intervals were completed. The mean value of the last two recordings was used in this article. Hypertension was defined as a systolic blood pressure ≥140 mm Hg, diastolic blood pressure ≥90 mm Hg, or current use of antihypertensive medication. Measurements of height and weight were performed using electronic scales, with participants wearing no shoes and light clothing. The body mass index (BMI) was calculated by dividing the weight in kilograms by the square of height in meters.

Information on diabetes and smoking was obtained from the questionnaires. The question on diabetes was stated as follows: “do you have or have you had diabetes?” (yes/no). Subjects were defined as non-smokers only if answering “no” to all three of the following questions: “do you smoke cigarettes daily?”; “do you smoke cigars/cigarillos daily?”; or “do you smoke pipe daily?” Study participants answering “yes” to any of the three were defined as daily smokers.

### Outcome Assessment

All first-time events of MI and ischemic stroke were identified by searching a wide range of International Classification of Disease (ICD) codes at the University Hospital of North Norway and the National Causes of Death Registry at Statistics Norway. The Norwegian national 11-digit identification number allowed linkage to both diagnosis and death registries. The University Hospital of North Norway is the only hospital serving the municipality of Tromsø, and the next nearest hospital is around 250 km away. This ensured that very few cases were lost during follow-up. An independent endpoint committee validated all stroke and MI events by retrieval of hospital medical records, death certificates, autopsy reports, and records from nursing homes, ambulance services, and general practitioners. The thorough process of identification and validation of outcomes has been previously described in detail. 6 9

### Statistical Analyses

Statistical analyses were performed with STATA version 14.0 (StataCorp LLC, College Station, Texas, United States). Follow-up time was calculated from the date of enrolment in Tromsø 4 (1994–1995) to the date of a stroke or MI event, date of death, date of migration from Tromsø, or until the end of follow-up (December 31, 2012), whichever came first. A total of 115 subjects experienced both outcomes during follow-up, and were censored from the first occurring event.

The study population was categorized into quintiles according to their baseline RDW values (Quintile 1: ≤12.4%, Quintile 2: 12.5–12.8%, Quintile 3: 12.9–13.1%, Quintile 4: 13.2–13.5%, Quintile 5: ≥13.6%) and their baseline hs-CRP values (Quintile 1: 0.01–0.51 mg/L, Quintile 2: 0.52–0.88 mg/L, Quintile 3: 0.89–1.47 mg/L, Quintile 4: 1.48–2.68 mg/L, Quintile 5: 2.69–9.97 mg/L). Age-adjusted baseline characteristics across quintiles of RDW and hs-CRP were estimated using analysis of covariance (ANCOVA). The correlation between RDW and hs-CRP was evaluated by calculating crude Pearson's correlation coefficients.

Crude incidence rates (IRs) were calculated as the total number of events divided by the total person time, and expressed as number of events per 1,000 person-years at risk. Cox proportional hazard regression models were used to estimate hazard ratios (HRs) with 95% confidence intervals (CI) for MI and ischemic stroke across quintiles of RDW and hs-CRP, and per 1 SD increment in RDW (0.8%) and hs-CRP (1.69 mg/L). Subjects in Quintile 1 of each exposure group were used as reference in the categorical analyses. Age was used as time scale in the regression analyses. The multivariable analyses were divided into three different models. Model 1 included adjustments for age (through the time scale), sex, and BMI. Model 2 included the covariates in model 1, in addition to smoking status, platelet count, hypertension, total cholesterol, triglycerides, and self-reported diabetes. In the analyses across quintiles of RDW, hs-CRP was added as a covariate to adjustment model 2, to evaluate whether chronic inflammation confounded the association between RDW and the CVD outcomes. In the analyses across quintiles of hs-CRP, baseline RDW values were added to adjustment model 2, to evaluate the potential mediating role of RDW. Furthermore, mediation analyses were conducted to calculate the proportion of the total exposure–outcome effect that could be due to the mediator. 25

## Results

There were 913 incident MIs (IR: 11.5 per 1,000 person-years, 95% CI: 10.7–12.2) and 461 incident ischemic stroke events (IR: 5.8 per 1,000 person-years, 95% CI: 5.3–6.3) during a median follow-up of 17.6 years. Table 1 shows crude baseline characteristics according to subjects who experienced MI, subjects who experienced ischemic stroke, and those who remained free of arterial CVD throughout the study period. The proportion of men, subjects with diabetes and hypertension, as well as age, levels of triglycerides, RDW, hs-CRP, hemoglobin, and BMI were all higher in those with than those without arterial CVD. The proportion of smokers was higher among those suffering an MI, but not among those with ischemic stroke. The crude correlation analysis revealed a low, but statistically significant correlation coefficient between hs-CRP and RDW ( *r* = 0.13, *p* < 0.0001). Age-adjusted baseline characteristics across quintiles of RDW and hs-CRP are shown in Supplementary Table S1 .

**Table 1:** Baseline characteristics stratified according to future development of myocardial infarction or ischemic stroke (The Tromsø Study)

	No event	Myocardial infarction	Ischemic stroke
*N*	4,506	913	461
RDW, %	13.0 ± 0.8	13.1 ± 0.8	13.2 ± 1.0
hs-CRP, mg/L	1.67 ± 1.69	2.11 ± 1.90	2.17 ± 1.86
Age, y	58.4 ± 10.5	63.8 ± 7.5	65.3 ± 6.8
Sex, % males	44.4 (2001)	59.5 (543)	53.8 (248)
Body mass index kg/m ^2^	25.7 ± 3.9	26.6 ± 4.1	26.4 ± 3.9
Daily smoking, %	32.0 (1,440)	38.2 (349)	31.0 (143)
Hemoglobin (total), g/dL	14.1 ± 1.1	14.4 ± 1.0	14.3 ± 1.1
Thrombocytes, ×10 ^9^ /L	248 ± 56	246 ± 56	247 ± 55
Hypertension, %	50.8 (2,288)	72.8 (665)	77.2 (356)
Total cholesterol, mmol/L	6.7 ± 1.3	7.0 ± 1.2	6.8 ± 1.3
Triglycerides, mmol/L	1.6 ± 1.0	1.9 ± 1.1	1.8 ± 1.0
Red blood cells, ×10 ^12^ /L	4.6 ± 0.4	4.7 ± 0.4	4.7 ± 0.4
Self-reported diabetes, %	1.9 (85)	5.6 (51)	6.5 (30)

Abbreviations: hs-CRP, high-sensitivity C-reactive protein; RDW, red cell distribution width.

Note: Baseline characteristics of study participants stratified according to future development of myocardial infarction or ischemic stroke. The values are reported as means ± standard deviation or as percentages with number in brackets.

Table 2 displays IRs and HRs for MI and ischemic stroke across quintiles of hs-CRP. The crude IRs of both MI and ischemic stroke increased with increasing serum concentrations of hs-CRP. In multivariable-adjusted analyses, subjects with hs-CRP in the upper quintile had a 44% higher risk of MI (model 2, HR: 1.44, 95% CI: 1.14–1.80) and 64% higher risk of ischemic stroke (model 2, HR: 1.64, 95% CI: 1.20–2.24) compared with subjects with hs-CRP in the lowest quintile. The risk estimate for MI was essentially unchanged after including RDW in the adjustment model (model 3, HR: 1.42, 95% CI: 1.13–1.78), whereas the risk estimate for ischemic stroke was modestly attenuated (model 3, HR: 1.59, 95% CI: 1.16–2.17). In mediation analyses, RDW was estimated to mediate 7.2% (95% CI: 4.0–30.8%) of the total association between hs-CRP and ischemic stroke. The association between hs-CRP and MI was not mediated by RDW (0.6%, 95% CI: −1.4 to 4.3%). In the analysis of CRP as a continuous variable, 1 SD (1.69 mg/L) increase in hs-CRP was associated with an 8% higher risk of MI (model 2, HR: 1.08, 95% CI: 1.04–1.12) and an 11% higher risk of ischemic stroke (model 2, HR: 1.11, 95% CI: 1.06–1.16).

**Table 2:** Adjusted HRs for myocardial infarction and ischemic stroke according to categories of hs-CRP (The Tromsø Study)

hs-CRP	Events	Crude IR (95% CI)	Age/Sex-adjusted HR (95% CI)	Model 1 HR (95% CI)	Model 2 HR (95% CI)	Model 3 HR (95% CI)
Myocardial infarction						
Q1	126	7.2 (6.0–8.6)	Ref.	Ref.	Ref.	Ref.
Q2	153	9.3 (8.0–10.9)	1.08 (0.85–1.36)	1.05 (0.83–1.33)	0.97 (0.77–1.24)	0.97 (0.76–1.23)
Q3	195	12.0 (10.4–13.8)	1.33 (1.06–1.67)	1.26 (1.00–1.58)	1.11 (0.88–1.39)	1.10 (0.87–1.39)
Q4	196	12.6 (10.9–14.5)	1.39 (1.11–1.74)	1.30 (1.04–1.63)	1.10 (0.87–1.39)	1.09 (0.86–1.38)
Q5	243	17.4 (15.4–19.8)	1.94 (1.56–2.40)	1.81 (1.45–2.25)	1.44 (1.14–1.80)	1.42 (1.13–1.78)
Per 1 SD increase	913	11.5 (10.7–12.2)	1.12 (1.09–1.16)	1.11 (1.08–1.15)	1.08 (1.04–1.12)	1.08 (1.04–1.11)
**Ischemic stroke**						
Q1	65	3.7 (2.9–4.7)	Ref.	Ref.	Ref.	Ref.
Q2	73	4.4 (3.5–5.6)	0.98 (0.70–1.37)	0.96 (0.69–1.35)	0.87 (0.62–1.23)	0.87 (0.62–1.22)
Q3	90	5.5 (4.5–6.8)	1.15 (0.84–1.59)	1.12 (0.81–1.55)	1.00 (0.72–1.38)	0.99 (0.71–1.37)
Q4	97	6.2 (5.1–7.6)	1.31 (0.95–1.79)	1.27 (0.92–1.74)	1.13 (0.82–1.57)	1.11 (0.80–1.54)
Q5	136	9.8 (8.2–11.5)	2.06 (1.53–2.77)	2.00 (1.48–2.70)	1.64 (1.20–2.24)	1.59 (1.16–2.17)
Per 1 SD increase	461	5.8 (5.3–6.3)	1.14 (1.09–1.19)	1.14 (1.09–1.19)	1.11 (1.06–1.16)	1.10 (1.05–1.16)

Abbreviations: BMI, body mass index; CI, confidence interval; hs-CRP, high-sensitivity C-reactive protein; HR, hazard ratio; IR, incidence rate; RDW, red cell distribution width; SD, standard deviation.

*Notes:*

Model 1: Age, sex, BMI.

Model 2: Model 1 + smoking, platelet count, hypertension, total cholesterol, triglycerides, self-reported diabetes.

Model 3: Model 2 + RDW.

IRs per 1,000 person-years and adjusted HRs with 95% CI for myocardial infarction and ischemic stroke across quintiles of hs-CRP, and per 1 SD increase in hs-CRP.

HRs and IRs for MI and ischemic stroke across quintiles of RDW are shown in Table 3 . In the multivariable model, subjects with RDW values in the highest quintile had a 22% higher risk of MI (HR: 1.22, 95% CI: 0.98–1.54) and a 44% higher risk of ischemic stroke (HR: 1.44, 95% CI: 1.06–1.97) compared with the reference group. After addition of hs-CRP to the multivariable analyses, the risk estimates were slightly attenuated for both outcomes, with a HR for MI of 1.17 (model 3, 95% CI: 0.93–1.47), and a HR for ischemic stroke of 1.37 (model 3, 95% CI: 1.00–1.87). When modeled as a continuous variable, a 1 SD increase (0.8%) in RDW was associated with a 6% higher risk of MI (model 2, HR: 1.06, 95% CI: 0.99–1.14) and a 14% higher risk of ischemic stroke (model 2, HR: 1.14, 95% CI: 1.05–1.24). These risk estimates were also slightly attenuated by adding hs-CRP to the multivariable analysis (model 3).

**Table 3:** Adjusted HRs for myocardial infarction and ischemic stroke according to categories of RDW (The Tromsø Study)

RDW	Events	Crude IR (95% CI)	Age/Sex-adjusted HR (95% CI)	Model 1 HR (95% CI)	Model 2 HR (95% CI)	Model 3 HR (95% CI)
Myocardial infarction						
Q1	138	8.0 (6.8–9.4)	Ref.	Ref.	Ref.	Ref.
Q2	198	9.5 (8.2–10.9)	1.01 (0.81–1.25)	1.00 (0.80–1.24)	0.99 (0.79–1.24)	0.98 (0.79–1.23)
Q3	184	12.6 (10.9–14.6)	1.23 (0.99–1.54)	1.22 (0.98–1.53)	1.19 (0.95–1.49)	1.18 (0.94–1.47)
Q4	190	13.8 (12.0–15.9)	1.30 (1.04–1.62)	1.29 (1.04–1.61)	1.22 (0.97–1.53)	1.20 (0.95–1.50)
Q5	203	15.4 (13.4–17.6)	1.41 (1.13–1.75)	1.40 (1.13–1.74)	1.22 (0.98–1.54)	1.17 (0.93–1.47)
Per 1 SD increase	913	11.5 (10.7–12.2)	1.11 (1.04–1.18)	1.11 (1.04–1.18)	1.06 (0.99–1.14)	1.04 (0.97–1.12)
**Ischemic stroke**						
Q1	70	4.0 (3.2–5.1)	Ref.	Ref.	Ref	Ref.
Q2	97	4.6 (3.8–5.7)	0.97 (0.71–1.32)	0.95 (0.70–1.30)	0.98 (0.71–1.33)	0.97 (0.71–1.32)
Q3	86	5.9 (4.8–7.3)	1.11 (0.81–1.53)	1.11 (0.81–1.52)	1.12 (0.81–1.54)	1.11 (0.80–1.53)
Q4	89	6.5 (5.3–8.0)	1.17 (0.86–1.61)	1.18 (0.86–1.61)	1.22 (0.88–1.69)	1.19 (0.86–1.64)
Q5	119	9.0 (7.5–10.8)	1.57 (1.17–2.11)	1.57 (1.16–2.11)	1.44 (1.06–1.97)	1.37 (1.00–1.87)
Per 1 SD increase	461	5.8 (5.3–6.3)	1.17 (1.09–1.26)	1.18 (1.09–1.27)	1.14 (1.05–1.24)	1.11 (1.03–1.21)

Abbreviations: BMI, body mass index; CI, confidence interval; hs-CRP, high-sensitivity C-reactive protein; HR, hazard ratio; IR, incidence rate; RDW, red cell distribution width; SD, standard deviation.

*Notes:*

Model 1: Age, sex, BMI.

Model 2: Model 1 + smoking, platelet count, hypertension, total cholesterol, triglycerides, self-reported diabetes.

Model 3: Model 2 + hs-CRP.

IRs per 1,000 person-years and adjusted HRs with 95% CI for myocardial infarction and ischemic stroke across quintiles of RDW, and per 1 SD increase in RDW.

## Discussion

In the present study, we explored the impact of chronic inflammation on the association between RDW and arterial CVD in a prospective, population-based cohort. RDW was associated with higher risks of MI and ischemic stroke, and these risk estimates were only modestly affected by adjustment for hs-CRP. Hs-CRP was associated with MI and ischemic stroke independent of other traditional atherosclerotic risk factors. Addition of RDW to the multivariable models slightly attenuated the association between hs-CRP and ischemic stroke, and specific mediation analyses indicated a modest role of RDW in mediating this relationship. Our findings suggest that chronic inflammation had only a modest impact on the relationship between RDW and arterial CVD.

CRP is a well-documented risk factor for arterial CVD. In a meta-analysis of 54 long-term prospective studies including 160,309 subjects without previous vascular disease, the risk ratio per 1 SD increase in CRP was 1.37 for coronary heart disease, and 1.27 for ischemic stroke. 17 Accordingly, we found an association between hs-CRP and both MI and ischemic stroke in our study. Furthermore, our results also concurred with most previous studies on the relationship between RDW and arterial CVD. In the Malmö Diet and Cancer study, a cohort with RDW measurements of 26,879 participants, Söderholm et al found a 30% higher risk of cerebral infarction for those in the highest versus the lowest RDW quartile. 8 In the same cohort, subjects in the highest RDW quartile had an 82% higher risk of fatal coronary events, but not increased risk of nonfatal coronary events (HR: 0.96, 95% CI: 0.82–1.12), compared with those in the lowest quartile. 7 In previous studies using the phase 1 population from Tromsø 4 ( *n* > 26,000), subjects with RDW in the highest quintile had 30% higher risk of ischemic stroke, 9 and 34% higher risk of MI compared with subjects in the lowest quintile. 6 Results from a prospective study on 3,226 previously healthy subjects from Taiwan, 26 however, reported no relationship between RDW and incident MI.

Several studies have reported a relationship between various markers of inflammation and RDW. Chronic diseases and inflammation affect the erythropoiesis and modulate red cell life span, and can thereby cause changes in the RDW. 18 In a cohort of 3,845 adult outpatients, Lippi et al found a threefold higher ESR and CRP in subjects with RDW in the highest compared with the lowest quartile. 19 A correlation between serum levels of CRP and RDW has been described in patients with non-dipper hypertension ( *r* = 0.403, *p* < 0.001), 27 patients with coronary artery disease ( *r* = 0.181, *p* < 0.001), 22 and in overweight adolescents ( *r* = 0.241, *p* = 0.034). 28 In patients with Alzheimer's disease, both CRP ( *r* = 0.350, *p* < 0.001) and ESR ( *r* = 0.457, *p* < 0.001) correlated with RDW. 29

Previously, only a few studies have investigated the potential impact of chronic inflammation as a confounder of the relationship between RDW and adverse outcomes. In the Malmö Diet and Cancer study, the association between RDW and fatal coronary events remained unchanged after addition of leukocyte count to the multivariable analyses. 7 Similarly, the association between RDW and heart failure did not change after adjustments for CRP in a large prospective study, 30 and a 1% increment in RDW yielded equal risk of all-cause mortality independent of hs-CRP levels. 31

Our findings undermine the hypothesis that chronic inflammation is the main explanation for the observed relationship between RDW and risk of arterial CVD. First, RDW and hs-CRP levels were weakly correlated in our population. Second, the relationship between RDW and arterial CVD was only modestly affected by adding hs-CRP to the multivariable analyses. However, residual confounding by inflammation cannot be completely ruled out, as our measurements of hs-CRP might not sufficiently reflect a chronic inflammatory state. Nevertheless, if the effect of RDW on arterial CVD is indirect, residual confounding by a factor other than inflammation seems more plausible in the light of our findings. Oxidative stress, stimulated by common cardiovascular risk factors, plays a crucial role in the pathogenesis of atherosclerosis. 32 33 Oxidative stress is also an important regulator of hematopoietic cell homeostasis, and could thereby influence RDW and confound the relationship between RDW and arterial CVD. 34 Unfortunately, we do not have measurements of reactive oxygen species in our population, and we were unable to adjust for this potential confounder. Anemia, shown to be a risk factor for CVD, 35 could also potentially confound the association, as it is clearly linked to RDW. However, several prospective studies, including previous analyses of the Tromsø study, have shown that the association between RDW and arterial CVD is independent of anemia. 6 8 9 36

A direct relationship between erythrocyte size variability and arterial CVD may be biologically plausible. Erythrocytes play an important role in atherosclerotic plaque development and stability. Deposition of the free cholesterol contained in the erythrocyte membrane is considered an important contributor to atherosclerotic plaque growth, 37 38 and atherosclerotic plaques susceptible to rupture tend to have a higher lipid content than stable plaques. 39 RDW is associated with a higher cholesterol content in the erythrocyte membranes, 40 and intraplaque hemorrhage could thereby have a greater impact on plaque growth and instability in subjects with high RDW. Furthermore, RDW is associated with reduced red cell deformability, 41 which again is linked to increased aggregation of erythrocytes, altered blood viscosity, and impaired blood flow in the microcirculation. 42 43 44 RDW is also associated with venous thromboembolism, a disease in which mechanism of hypercoagulability and stasis are central players. Our results point toward a modest and direct role of RDW in the development of ischemic stroke, but not in the development of MI. Growing evidence supports more pronounced roles for hypercoagulability and stasis in the pathogenesis of ischemic stroke than in the pathogenesis of MI. First, mechanisms related to hypercoagulability, such as prothrombotic genotypes, are reported to be more pronounced in ischemic stroke than in MI. 45 Second, approximately 40% of the ischemic strokes are caused by cardioembolic events, in which stasis plays a pivotal role in the pathogenesis. 46

A major strength of our study is the prospective design with a long follow-up time. Participants were recruited from the general population with a high attendance rate. Few cases were missed during follow-up due to the single hospital serving the Tromsø area, and the thorough process of case identification and validation. One limitation to the study is the skewed age distribution due to the selected population invited to the second visit of Tromsø 4, which may reduce the generalizability of our findings. There was only one measurement of hs-CRP and RDW throughout the study period, which could result in an underestimation of the true associations due to regression dilution bias. 47 Another potential weakness with only one measurement is the uncertainty of whether increased hs-CRP levels could be due to a chronic or an acute inflammatory process. To avoid this, all subjects with hs-CRP >10 mg/L were excluded from our analyses, as this indicates an ongoing acute-phase response. 48

In conclusion, we found that the association between RDW and arterial CVD was not confounded by hs-CRP, and that RDW had a modest direct effect on the risk of ischemic stroke, but not MI. Our study calls for a reassessment of chronic inflammation as the main explanation for the association between RDW and arterial CVD. Future studies are warranted to further explore other potentially underlying mechanisms.
