# Supplementary material for: Impact of Chronic Inflammation, Assessed by hs-CRP, on the Association between Red Cell Distribution Width and Arterial Cardiovascular Disease: The Tromsø Study
Source: TH Open. 2018 May 16;2(2):e182–9. doi: 10.1055/s-0038-1651523 (PMC6524874; doi:10.1055/s-0038-1651523)
Supplement: Supplementary file 1 — Supplementary Table S1 [file 10-1055-s-0038-1651523-s180008.pdf]

Supplementary Table S1 Age-adjusted baseline characteristics across quintiles of RDW and hs-CRP: The Tromsø Study

	RDW					hs-CRP				
	1	2	3	4	5	1	2	3	4	5
N	1,185	1,441	1,049	1,007	1,085	1,178	1,131	1,154	1,159	1,145
Range	11.0–12.4	12.5–12.8	12.9–13.1	13.2–13.5	13.6–25.2	0.01–0.51	0.52–0.88	0.89–1.47	1.48–2.68	2.69–9.97
Age, y	56.2 ± 11.9	58.9 ± 9.8	60.5 ± 9.4	61.0 ± 9.3	62.4 ± 9.2	56.4 ± 11.6	59.1 ± 10.4	60.5 ± 9.4	61.1 ± 9.1	61.3 ± 9.7
Sex, % males	39.7 (470)	45.2 (651)	48.7 (511)	51.9 (523)	52.7 (572)	40.6 (478)	48.4 (547)	47.0 (542)	48.9 (567)	51.7 (592)
Body mass index, kg/m^2	25.7 ± 3.7	26.0 ± 3.7	26.1 ± 4.0	25.8 ± 4.0	25.7 ± 4.2	24.4 ± 3.1	25.2 ± 3.4	26.1 ± 3.8	26.8 ± 4.1	27.0 ± 4.3
Daily smoking, %	19.9 (236)	26.6 (383)	33.4 (350)	40.8 (411)	47.8 (519)	24.4 (287)	27.8 (314)	33.2 (383)	36.2 (420)	43.3 (496)
Hemoglobin, g/dL	14.2 ± 1.1	14.2 ± 1.0	14.3 ± 1.0	14.2 ± 1.1	13.9 ± 1.2	13.9 ± 1.1	14.1 ± 1.1	14.2 ± 1.0	14.2 ± 1.1	14.3 ± 1.1
Thrombocytes, $\times10^9$/L	242 ± 50	247 ± 52	246 ± 51	249 ± 54	256 ± 73	239 ± 52	247 ± 55	246 ± 59	250 ± 53	257 ± 61
Hypertension, %	56.7 (672)	55.3 (797)	57.3 (601)	52.3 (527)	56.8 (616)	48.8 (575)	52.7 (596)	58.3 (673)	56.4 (654)	62.5 (716)
Total cholesterol, mmol/L	6.6 ± 1.3	6.7 ± 1.3	6.8 ± 1.3	6.8 ± 1.3	6.7 ± 1.3	6.6 ± 1.3	6.7 ± 1.3	6.7 ± 1.3	6.8 ± 1.3	6.8 ± 1.2
Triglycerides, mmol/L	1.7 ± 1.1	1.7 ± 1.1	1.7 ± 1.1	1.6 ± 0.9	1.6 ± 0.9	1.4 ± 0.9	1.5 ± 1.0	1.7 ± 1.0	1.8 ± 1.1	1.9 ± 1.1
Red blood cells, $\times10^{12}$/L	4.6 ± 0.4	4.7 ± 0.4	4.7 ± 0.4	4.7 ± 0.4	4.6 ± 0.4	4.6 ± 0.4	4.6 ± 0.4	4.7 ± 0.4	4.7 ± 0.4	4.7 ± 0.4
Self-reported diabetes, %	3.1 (37)	3.3 (48)	3.1 (33)	1.6 (16)	1.9 (21)	1.1 (13)	2.2 (25)	2.3 (27)	2.6 (30)	5.0 (57)
RDW, %	–	–	–	–	–	12.9 ± 0.7	13.0 ± 0.8	13.0 ± 0.9	13.1 ± 0.8	13.2 ± 1.0
hs-CRP, mg/L	1.53 ± 1.62	1.65 ± 1.65	1.63 ± 1.61	1.86 ± 1.70	2.22 ± 2.02	–	–	–	–	–

Notes: Age-adjusted baseline characteristics of study participants across quintiles of red cell distribution width (RDW) and high-sensitivity C-reactive protein (hs-CRP). The values are reported as means ± standard deviation or as percentages with number in brackets.
